# Empirical Estimation of *R*_0_ for Unknown Transmission Functions: The Case of Chronic Wasting Disease in Alberta

**DOI:** 10.1371/journal.pone.0140024

**Published:** 2015-10-09

**Authors:** Alex Potapov, Evelyn Merrill, Margo Pybus, Mark A. Lewis

**Affiliations:** 1 Department of Biological Sciences, University of Alberta, Edmonton, AB, T6G 2G1, Canada; 2 Centre for Mathematical Biology, University of Alberta, Edmonton, AB, T6G 2G1, Canada; 3 Department of Mathematical and Statistical Sciences, University of Alberta, Edmonton, AB, T6G 2G1, Canada; 4 Alberta Sustainable Resource Development, 6909–116 St., Edmonton, AB, T6H 4P2, Canada; University of Florida, UNITED STATES

## Abstract

We consider the problem of estimating the basic reproduction number *R*_0_ from data on prevalence dynamics at the beginning of a disease outbreak. We derive discrete and continuous time models, some coefficients of which are to be fitted from data. We show that prevalence of the disease is sufficient to determine *R*_0_. We apply this method to chronic wasting disease spread in Alberta determining a range of possible *R*_0_ and their sensitivity to the probability of deer annual survival.

## Introduction

The basic reproduction number [1], *R*_0_, is one of the well-known epidemiological characteristics. It quantifies the average number of secondary cases per infected individual in the beginning of a disease outbreak. It’s most common use is to determine vaccination level needed to stop disease spread. In the simplest case, when sex, age and social structure are not important, and the rate of a disease spread depends only on the total number of infected individuals, *R*_0_ equals to the mean number of new infections per a currently infected individual in a totally susceptible population. If a part of the population is vaccinated, some of these *R*_0_ individuals do not develop the infection. As a result, the disease spread slows down. For example, if *R*_0_ = 4, then the disease would not spread if 3 out of 4 individuals are immune; that is, at least 1 − *R*_0_^−1^ = 75% of individuals have to be vaccinated and develop an immune response.

Generalization of *R*_0_ for the case where there are several categories of infected individuals with differences in survival or disease spread rate, has been suggested by Diekmann et al. [2]. Here the basic reproduction number describes the ratio of the total number of individuals in two successive generations provided the proportions of infected individuals of all types correspond to the so-called the stable composition, a mixture of infection types that one would expect to see if the disease were allowed to grow at low levels for many generations. Diekmann et al. [2] describes this as the “typical number of secondary cases per infected individual”. Mathematically, this definition of *R*_0_ is equivalent to the spectral radius (maximum eigenvalue) of the next-generation operator and the stable distribution is given by the associated eigenvector.

In some instances, there is a problem of backward bifurcation [3], where disease can break out even when *R*_0_ < 1. This is possible for certain kinds of dynamics, providing the infectives are introduced at levels sufficient to induce nonlinear feedbacks. However, in numerical studies of Chronic Wasting Disease, to which we apply our analysis, a backward bifurcation has not been observed. Therefore it is not envisaged in our analysis, but is discussed further in the Discussion section

We present a generalization of the method for calculating *R*_0_ from the intrinsic growth rate of infection in a population [1] for the case where there are multiple infectious compartments reflecting individual age, sex and perhaps other characteristics. Similar to the method of intrinsic growth rate, we do not assume any specific disease transmission mechanism (e.g. density or frequency-dependent), but approximate the matrix of marginal force of infection from observed data. This matrix plays the role of the intrinsic growth rate. The second novelty is that we base our approach upon the equation for the change of the disease prevalence over time. The resulting model is linear and calculation of *R*_0_ is then done by standard methods. Our approach is useful when the mechanisms of disease transmission are incompletely understood or too complicated to fully model. However, it relies on a record of the growth in prevalence during the early stages of the disease outbreak when the prevalence is close to zero.

We apply the method to estimate *R*_0_ for chronic wasting disease (CWD), a slowly spreading prion disease of cervids for which there is limited information about the exact mechanisms of host transmission, but where data about the growth of disease prevalence were collected from mandatory surveillance in Alberta programs over the past 8 years. CWD was first detected in the wild in a mule deer in Saskatchewan (SK) near the Saskatchewan-Alberta border in 2000. In Alberta 210 infected cases, primarily mule deer, have been detected since 2005 in the Battle River and Red Deer/South Saskatchewan River drainages [4]. Our previous publications [5,6] focused on developing models of CWD transmission and harvest management. Here we partially relied on previously published transmission models [6] as one of the approaches to reduce the number of parameters in the problem of *R*_0_ estimation. In this paper, we simplify these models and fit them to early growth of the disease so as to obtain a range of possible *R*_0_ values. The obtained values of *R*_0_ may allow managers to evaluate risk of each infected individual for evaluating control strategies even during the early stages of the disease outbreak. We note that models for CWD in cervids are considered in a number of papers, e.g. [7,8,9,10,11]. However, due to complexity of the processes involved, a reliable parameterization of a more or less detailed model has not been possible. For this reason, here we consider a very basic linear model, fit it to available data, and then estimate *R*_0_.

## Theory

Disease models typically include several compartments indicating disease status (e.g., susceptible, infected, exposed individuals), which in turn could be subdivided into categories of population structure such as sex or age. Below, we only keep track of infected individuals, assuming that 1) all categories of susceptible individuals are at disease-free equilibrium *S*_0*k*_ and 2) the model including only susceptible and infected individuals can effectively describe the disease dynamics even if the disease has an exposed (or latent) stage [6,12]. So, susceptible individuals are implicitly present in the model.

### General assumptions about the disease transmission function

Consider the case with *n* types of infected individuals, describing sex and age classes. If we denote the number of infected individuals in class *k* at time *t* by *I*_*k*_(*t*), then the disease prevalence in the *k*-th type at time *t* is *i*_*k*_(*t*) ≈ *I*_*k*_(*t*)/*S*_0*k*_ << 1. Growth in the number of the *k*-th type of infected individuals is described by a force-of-infection function *f*_*k*_(*i*_1_,…,*i*_*n*_), which is unknown. Individuals leave the infected class at a per capita rate *m*_*k*_ due to mortality or recovery or both, which are equivalent within the frame of our approach. For CWD, there is no recovery class, so we use the terms mortality and survival rate only. We assume that, at low prevalence, the transmission function is proportional to *i*_*k*_(*t*), which is typical of the most commonly used transmission functions. In other words, we exclude dependencies having the leading terms like *i*_*k*_^*q*^, *q* ≠ 1; see an example in [13] for the case 0 < *q* < 1. Because there is no transmission in the absence of the disease, *f*_*k*_(0,…,0) = 0. For *i*_*k*_ << 1, higher order terms *i*_*k*_^2^, *i*_*k*_ *i*_*m*_,… become negligible, and the linear approximation of the force of infection suffices:

$$f_{k}\left(i_{1},\ldots ,i_{n}\right)\approx F_{k1}i_{1}+F_{k2}i_{2}+\ldots +F_{kn}i_{n}=\sum_{j=1}^{n}F_{kj}i_{j},$$ (1)

where *F*_*kj*_ describes the disease transmission from individuals of type *j* to type *k*.

It is possible to construct both continuous-time and discrete-time models of the disease-prevalence growth, either of which can be used to calculate *R*_0_. The choice of model is a matter of convenience, and should most probably be determined by whether mortality rate or per-year survival is available.

We consider the case when infected individuals do not change their compartment; that is, they only become infected due to the force of infection, and then die or become uninfected. This is true, for example, when there is no age structure for the infected individuals, juveniles are practically noninfected, or the disease duration is short, such that change of age class of infected individuals (juvenile/adult) can be neglected. In the end of this section, we discuss the changes in the approach if age structure is necessary.

### Discrete-time model

Here we derive a linear matrix model for the disease prevalence and show that it alone is sufficient to obtain *R*_0_. In the case of the discrete-time model with time step *τ*, mortality is described by per-time-step survival probability of infected individuals, *s*_*Ik*_, and healthy individuals, *s*_*Hk*_. The latter appears in the term of new infections where in most cases it is reasonable to assume that, during the first time step, mortality of newly infected individuals should be the same as that of the healthy ones. The dynamics of the infected compartment is described by the equation

$$I_{k}\left(t+\tau \right)=\left(1-\exp\left(-\tau f\left(i_{1},\ldots ,i_{n}\right)\right)\right)s_{Hk}S_{k}\left(t\right)+s_{Ik}I_{k}\left(t\right).$$ (2)

Using approximation (1) and taking into account that, for 0 ≤ *x* << 1, 1 – *e* ^−*x*^ ≈ *x*, we obtain the following model for the number of infected individuals:

$$I_{k}\left(t+\tau \right)=\tau s_{Hk}S_{0k}\sum_{j=1}^{n}F_{kj}i_{j}+s_{Ik}I_{k}\left(t\right),$$ (3)

Or, for the prevalence,

$$i_{k}\left(t+\tau \right)=\tau s_{Hk}\sum_{j=1}^{n}F_{kj}i_{j}+s_{Ik}i_{k}\left(t\right).$$ (4)

This equation allows us to fit *F*, which we call the matrix of “marginal force of infection”, from the data on change in the observed disease prevalence. However, to estimate the basic reproduction number, which is the number of new infections per one typical individual, we need to operate in terms of the number of individuals rather than prevalence. To connect these two quantities we introduce the diagonal matrices of both numbers of susceptible individuals and per time step survival probabilities. Denoting

$$D=\text{diag}\left(S_{01},\ldots ,S_{0n}\right),\;S_{H}=\text{diag}\left(s_{H1},\ldots ,s_{Hn}\right),\;S_{I}=\text{diag}\left(s_{I1},\ldots ,s_{In}\right),$$ (5)

and using the matrix *D* to relate prevalence and the number of infected individuals, *I* = *Di* or *i* = *D* ^−1^ *I*, it is possible to write a simplified form of Eq (3) in matrix form as

$$I\left(t+\tau \right)=\left(\tau S_{H}DFD^{-1}+S_{I}\right)I\left(t\right).$$ (6)

The basic reproduction number *R*_0_ is the spectral radius *ρ*(⋅) [2,14,15] or the maximum eigenvalue of the next-generation matrix *G*,

$$R_{0}=\rho \left(G\right),\;G=\tau S_{H}DFD^{-1}{\left(1-S_{I}\right)}^{-1},$$ (7)

[16]. However, it is simple to show that eigenvalues of the matrices *M* = *τS*_*H*_ *F*(1 − *S*_*I*_)^−1^ and *G* coincide provided *D* ^−1^(1 – *S*_*I*_)^−1^ = (1 − *S*_*I*_)^−1^ *D* ^−1^, *S*_*H*_ *D* = *DS*_*H*_, which is always true if the matrices are diagonal. Since in this case

$$G=D\left(\tau S_{H}F{\left(1-S_{I}\right)}^{-1}\right)D^{-1}=DMD^{-1},$$ (8)

*G* **u** = *λ* **u** implies *M* **v** = *λ* **v** where **v** = *D* ^−1^ **u**, and hence

$$R_{0}=\rho \left(M\right),\;M=\tau S_{H}F{\left(1-S_{I}\right)}^{-1}.$$ (9)

Note that we do not need to know the matrix *D* and the equation for the prevalence (4) is enough to obtain *R*_0_. Therefore, if the matrix of marginal force of infection *F* can be estimated from data by fitting model (4), calculation of the basic reproduction number is straightforward. Survival matrices *S* (5) must be obtained elsewhere.

The continuous-time model can be considered in a similar way (see Appendix A1 in S1 File). It also is possible to show the equivalence of both approaches (Appendix A1 in S1 File): for small time steps *τ* (9) turns into the relation for the continuous-time case. Therefore both continuous and discrete time approaches are equivalent, and the choice of discrete or continuous time model is a matter only of preference.

### Meaning of matrices *M* and *G* = *DMD* ^−1^

The next-generation matrix *G* can be called the matrix of secondary infections, because its entry *G*_*ij*_ is the number of secondary infections of type *i* produced by one infected individual of type *j* during its lifetime (see e.g. [14,17]). Therefore the sum of the entries in column *j*,

$$q_{Gj}=G_{1j}+G_{2j}+\ldots +G_{nj},$$ (10)

is the total number of new infections produced by one infected individual of type *j* during its lifetime. It is sometimes true that *q*_*Gj*_ may be greater than *R*_0_. That is, if we have only one infected individual of this type, in the next generation the number of infections will be greater than *R*_0_. However, after many generations, when the proportions of infected individuals of different classes stabilize, the number of new infections will grow as *R*_0_.

Consider a simple example. Let there be two types of infected individuals—e.g. males (*i* = 1) and females (*i* = 2) of some species—and

$$G=\left(\begin{array}{cc} 2 & 4\\ 0 & 3 \end{array}\right).$$ (11)

(Instead of zero there may be a small number, but conclusions remain the same.) Here *R*_0_ = 3, but *q*_*G*1_ = 2 and *q*_*G*2_ = 7. Such an asymmetry may arise because, for example, one sex may infect the other during mating, but not vice versa. Therefore one male leaves behind two infected males, while one female infects 4 males and 3 females. If infection starts from one infected female—that is, with the state (0,1)—then the first 4 generations look as follows

(0,1)→(4,3)→(20,9)→(76,27)→(260,81)→…

After 6 generations, the ratio of infected males to females becomes about 3:1, and when it reaches 4:1 then at the next generation the ratio is 12:3 = 4:1. That is, this proportion reproduces itself exactly at the rate *R*_0_. Note that this description in terms of generations of infected individuals may differ from the dynamics of the total number of infected since the generations often overlap in time. At any given moment there may be infected individuals belonging to several generations.

In practical management, when short-term goals are considered, other characteristics can be important as well. For example, if the management goal is to minimize the number of infections in the next generation, it would be optimal to concentrate on removal of infected individuals with the maximum *q*_*Gj*_, which in the example above is infected females.

In contrast with *G*_*ij*_, which is the number of infected individuals in successive generations, *M*_*ij*_ relates prevalence in the next generation of infected individuals of type *i* to the current generation of infected individuals of type *j*. In general, it does not make sense to sum up prevalence for different types of individuals, and therefore it is hard to define analogs of *q*_*Gj*_ for *M*. The relation between *G* and *M* depends on population proportions of individuals of different types, *G*_*ij*_ = (*S*_0*i*_/*S*_0*j*_)*M*_*ij*_; therefore only weighted sums of *M* entries make sense.

### Non-diagonal mortality or survival matrix

The above analysis was done for the case of diagonal mortality matrix *V* or survival matrix *S*, when infected individuals cannot change their compartment except for leaving the infected state. For age-structured models, when infected individuals may change their age class, in general *G* ≠ *DMD* ^−1^ and eigenvalues of *M* and *G* may be different. Then the population structure becomes important for the disease dynamics and, like the calculation of *q*_*G*_, information about population proportions is necessary. Fitting a model based on disease prevalence still gives a marginal force of infection *F*, but calculation of *R*_0_ now requires the matrix *DFD* ^−1^, whose entries are *F*_*kj*_ × (*S*_0*k*_ / *S*_0*j*_). The mortality/survival matrix in the equation for prevalence also changes to *D* ^−1^ *VD* or *D* ^−1^ *S*_*I*_ *D*. For deer, one needs to know population ratios like buck:doe and fawn:doe to estimate *R*_0_.

### Fitting the marginal force of infection matrix *F* from the observed data

The specific algorithm of fitting depends on the nature of the data. In our case, we have a sample from the deer population provided by hunters, where we know the number of positive and negative cases, and sex of each deer. We used a stochastic model of population harvest (see Appendix A2 in S1 File) and maximum likelihood estimates of model parameters to derive AIC-based selection models.

Note that survival probabilities or mortality rates could be obtained from separate studies. Nonetheless, the number of model parameters to be fitted is still greater than the size of the matrix *F*. First, it is necessary to fit the prevalence at the initial moment. Second, the specific tasks may require accounting for management actions and/or immigration or emigration of infected individuals, which also creates additional parameters. If the amount of data is insufficient for estimating all the parameters, or there are other problems with parameter estimability, it may be necessary to simplify the model. This can be done with the help of simplifying hypotheses, both empirical and mechanistic. Then methods of statistical model selection may be very helpful for obtaining the results. An example of estimating the basic reproduction number in such a situation is presented in the next section.

## Application to CWD in Mule Deer in Alberta

### 3.1 Methods and data

In Alberta, ongoing voluntary submission of hunter-harvested deer heads for testing on CWD began in 1998. Mandatory head submission began in 2006 within an area of CWD risk that increased from 131,000 km^2^ in 2006 to 565,900 km^2^ in 2010–2012. The area of mandatory testing seems to be large enough to cover the affected area during this study. We assume that CWD prevalence in hunter-harvested deer is equal or proportional to that in the wild population. At present, there is no agreement whether a hunter harvest prevalence estimate is biased or non-biased. However, in our case, for estimating *R*_0_, proportionality is enough. It means that the prevalence in harvested individuals and in the population is related as *i*_*k*,harvest_ = *B*_*k*_ *i*_*k*_, provided the coefficients *B*_*k*_ do not change with time (the non-biased case corresponds to *B*_*k*_ = 1). Then it can be shown that, in the case of diagonal survival matrices, *i*_*k*,harvest_ leads to the same *R*_0_ estimate even if the bias is different for each deer category (when all *B*_*k*_ are equal, the diagonality of survival matrices is not necessary).

The complication of modeling CWD prevalence in Alberta is related to two things. First, in 2005–2008, a winter herd-reduction program was implemented with the goal of removing all deer within 10-km circles around the location of each fall hunter-harvested infected deer. In these open Prairie and Parkland areas, a total of over 7,000 deer were removed during winter disease-control programs. At the same time, there may be a continuing inflow of infected individuals from Saskatchewan, most probably young males because they are more likely to disperse [18,19,20]. Indeed, Nobert [21] found newly detected CWD positive cases in deer were influenced by their connectivity of sources in Saskatchewan. We adapted the above models to account for these influences.

Alberta CWD surveillance data includes the number of CWD-positive and negative males and females killed by hunters each year in 2006–2011 (Table 1) and in the herd-reduction program in 2006–2008 (Table 2) but do not report their age. Therefore only two classes of infected individuals (*n* = 2), males and females could be considered. We index *k* = 1 for males and *k* = 2 for females; hence matrix *F*_*ki*_ is a 2 × 2 matrix.

**Table 1:** Hunter harvest and CWD prevalence estimates for 2006–2011.

year	Male negative	Male positive	male prevalence	female negative	female positive	female prevalence
2006	727	2	0.0027	1059	2	0.0019
2007	1252	5	0.0040	1923	1	0.00052
2008	1184	6	0.0050	1455	1	0.00069
2009	1104	9	0.0081	1488	3	0.0020
2010	1450	13	0.0089	1471	5	0.0034
2011	865	18	0.0208	1060	12	0.0113

**Table 2:** Cull data for adult mule deer and comparison of prevalence in cull and hunter harvest animals. Two numbers marked with bold show very high ratio of CWD prevalence in culled and hunter-harvested animals.

year	male neg/pos	male prev., cull	male prev., hunt	male cull/ hunt	female neg/pos	female prev., cull	female prev., hunt	female cull/ hunt
2006	509 / 5	0.00973	0.00274	3.5	651 / 4	0.0061	0.00189	3.2
2007	190 / 4	0.02062	0.00398	5.2	315 / 6	0.0187	0.00052	**36.0**
2008	350 / 11	0.03047	0.00504	6.0	484 / 4	0.0082	0.00069	**11.9**

Annual survival rates for healthy mule deer in eastern Alberta were taken from Merrill et al. [22], where *s*_*H*1_ = 0.58 (males) and *s*_*H*2_ = 0.87 (females). Survival rates of infected deer in Alberta have not been measured directly. As a result, we used data from Miller et al. [23] indicating that the survival rate for infected mule deer females decreases by a factor of 0.64 (from 0.82 to 0.53), such that *s*_*I*2_ = 0.87 × 0.64 = 0.56; we assumed equal survival reduction for males where *s*_*I*1_ = 0.58 × 0.64 = 0.37.

Immigration of infected deer from outside Alberta requires the inclusion of an additional term. If there were a positive annual difference between the number of immigrated and emigrated infected individuals, Δ*I*, this would mean an increase in prevalence by *j* = Δ*I* / *S*_0_. We assume these values are constant across years.

The influence of the herd-reduction program can be estimated in a similar way. If, in year *t*, Δ*I*_*C*_ individuals were removed by culling programs, this would result in a prevalence reduction of Δ*i* = Δ*I*_*C*_ / *S*_0_ = *γ*Δ*I*, *γ* = 1 / *S*_0_. However, because the numbers of susceptible males and females are unknown, the coefficients *γ*_*k*_ should be fitted from the data as well.

The full model to be fitted to data is as follows:

$$i_{1}\left(t+1\right)=\tau s_{H1}F_{11}i_{1}\left(t\right)+\tau s_{H1}F_{12}i_{2}\left(t\right)+s_{I1}i_{1}\left(t\right)+j_{1}-\gamma_{1}\Delta I_{C1}\left(t\right),$$ (12)

$$i_{2}\left(t+1\right)=\tau s_{H2}F_{21}i_{1}\left(t\right)+\tau s_{H2}F_{22}i_{2}\left(t\right)+s_{I2}i_{2}\left(t\right)+j_{2}-\gamma_{2}\Delta I_{C2}\left(t\right).$$ (13)

The model contains up to 10 unknown parameters; to obtain the estimate of the basic reproduction number, we reduce this number down to 4−7 using simplifying hypotheses and model selection. The full set of parameters includes the matrix *F*_*ki*_, net immigration rates *j*_*k*_, culling factors *γ*_*k*_, and initial prevalence *i*_*k*_(2005). Because the surveillance data provide the number of positive and negative cases for males and females for 6 years—that is, only 24 numbers—we assume that either some *j*_*k*_ or *γ*_*k*_ are zero, or that the matrix *F* has a special structure or a fixed proportion in the initial data. We compare the models resulting from each of the assumptions with the help of AIC and AIC_C_. In the latter case, we estimate the correction term for *n* = 24 data points.

Fitting the model to data was done using maximum likelihood; standard error was estimated from Fisher information. Statistical models for the likelihood of the number of positive cases given prevalence and the total number of harvested individuals is in Appendix A2 in S1 File. The models were compared by likelihood estimates, and AIC and AIC_C_ values. To improve the accuracy of *R*_0_ estimates, in some cases we have chosen *R*_0_ as an estimated parameter instead of one of *F* entries. The idea of the approach is as follows: for example, we have a 2 × 2 matrix with the entries *a*,*b*,*c*,*d* and the largest eigenvalue *λ*. They are related by *bc* = (*λ* − *a*)(*λ* − *d*). Assuming *c* is nonzero, we can express *b* through *λ* and estimate parameters *a*,*λ*,*c*,*d*.

### 3.2 Results

**Fig 1:**
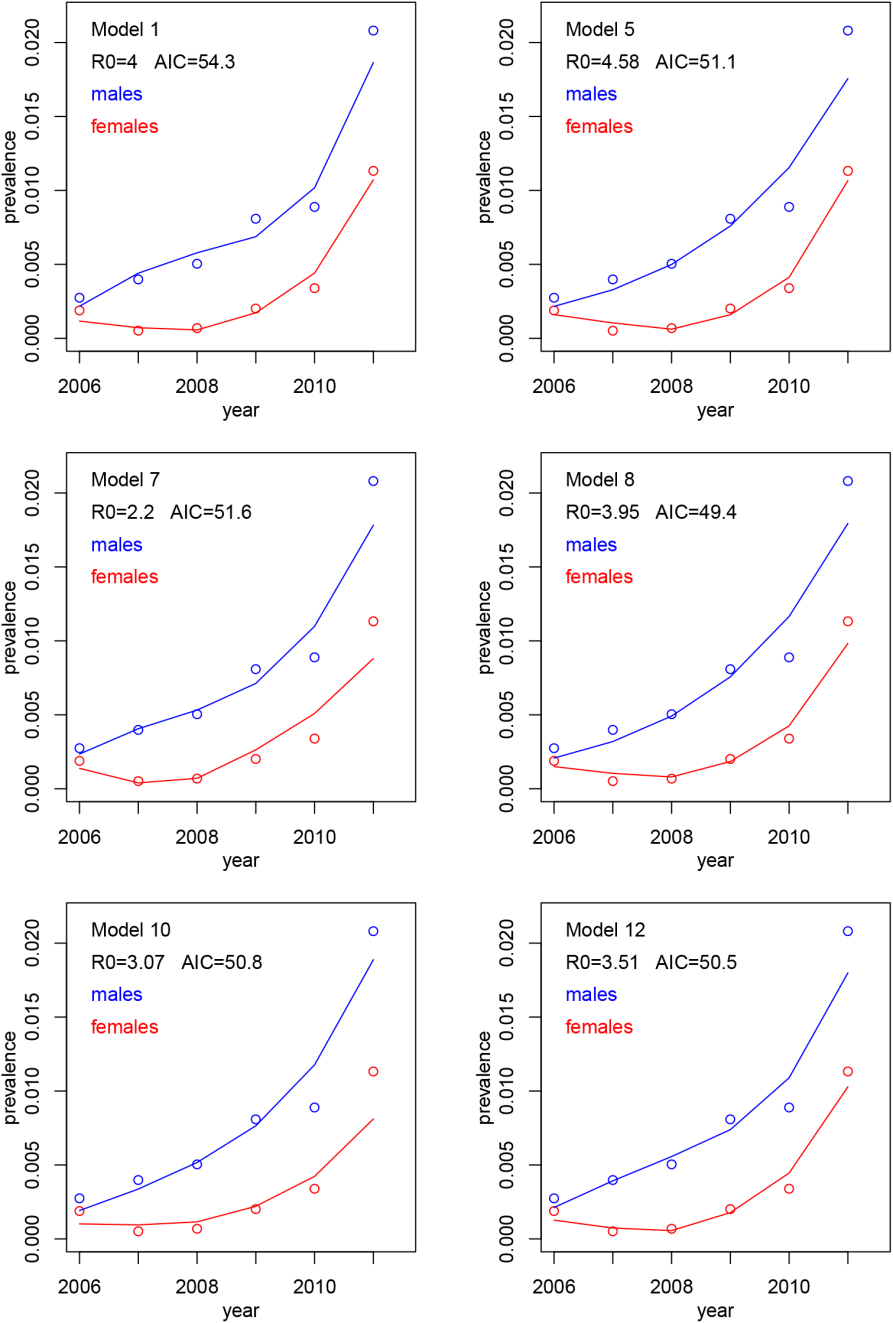
Circles show CWD prevalence in Alberta from hunter-harvest data in Table 1. Solid lines show Eqs (12) and (13) fit to the data. Various models correspond to different hypotheses about “fecundity” matrix *F* and are explained in Table 4.

Observed CWD prevalence for all deer sampled in 2006–2011 and the trajectory of the fitted model are plotted in Fig 1. We attempted to fit models (12) and (13) with different combinations of culling *γ*_1_,*γ*_2_ and net immigration *j*_1_,*j*_2_ terms to determine which of them are essential for assessing model fit (Table 3). The lowest AIC and AIC_C_ supported by the model are in line 12 of the table, which includes a culling term only for females *γ*_2_ ≈ 3.4 × 10^−4^ and has *R*_0_ ≈ 4.0. The corresponding matrix *F* was

$$F\approx \left(\begin{array}{cc} 1.1 & 3.3\\ 0.1 & 2.0 \end{array}\right).$$ (14)

However, the standard error exceeded values of the parameters. This happens because the profile of log-likelihood is close to flat in some directions and large changes of parameters along them lead to small changes in likelihood. To find these directions, we found eigenvalues and eigenvectors of the Hessian matrix at the point of likelihood maximum. There were two small eigenvalues, and the direction of the one of the eigenvectors almost exactly coincided with the initial prevalence for males, while the second eigenvector was a mixture of all parameters with the weights between 0.1 and 0.6, and did not allow for a simple interpretation.

**Table 3:** Fitting 6 to 10 parameter models to data, with culling terms *γ*_1_,*γ*_2_ and immigration terms *j*_1_,*j*_2_ present (+) or absent (–); see Eqs (12) and (13). The model in row 12 with the lowest AIC and AIC_C_ is shown in bold; it shows no culling effect for males. AIC_C_ also supports model in row 16 with no culling effect for either males or females. None of the best models show significance of immigration terms.

	*γ*_1_,*γ*_2_	*j*_1_,*j*_2_	AIC	AIC_C_	*R*_0_
1	+,+	+,+	62.2	79.1	3.95
2	+,+	+,–	60.2	73.1	2.78
3	+,+	–,+	60.2	73.1	2.63
4	+,+	–,–	58.2	67.8	4.58
5	+,–	+,+	64.6	77.5	3.05
6	+,–	+,–	62.6	72.2	3.04
7	+,–	–,+	62.7	72.3	3.02
8	+,–	–,–	60.7	67.7	2.98
9	–,+	+,+	60.3	73.2	3.67
10	–,+	+,–	58.3	67.9	4.37
11	–,+	–,+	58.3	67.9	3.64
**12**	**–,+**	**–,–**	**56.3**	**63.3**	**3.89**
13	–,–	+,+	62.6	72.2	3.05
14	–,–	+,–	60.6	67.6	3.03
15	–,–	–,+	60.7	67.7	3.02
16	–,–	–,–	58.7	**63.6**	**3.03**

To avoid the problem of estimating the initial prevalence, we fixed 7 different male:female ratios in 2005 as 1:0, 3:1, 2:1, 1:1, 1:2, 1:3 and 0:1, which reduces the number of initial prevalence parameters from 2 to 1. These ratios may be considered as alternative hypotheses about the initial disease state; we tested all of them and chose one with the least AIC.

Hypotheses about the initial state solved the problem with estimability of the initial prevalence, but the second dimension of flatness in parameter space still did not allow us to obtain reasonable error estimates (Table 4, row 1). For this reason, additional hypotheses about the matrix *F*—that is, about CWD transmission—were necessary.

**Table 4:** Testing hypotheses on the details of disease transmission (structure of the matrix *F* in Eqs (12) and (13)). Hypotheses with ΔAIC < 2 are marked with bold font. The same four models have the lowest AIC_C_ as well.

#	*F* type	Hypothesis	*R*_0_±se	AIC	AIC_C_	*F*	*γ*_2_ × 10^−4^
8	$\left(\begin{array}{cc} a & 0\\ 0 & a \end{array}\right)$	No f↔m	3.95±0.41	**49.4**	**50.6**	$\left(\begin{array}{cc} 2.01 & 0\\ 0 & 2.01 \end{array}\right)$	4.0±1.0
12	$\left(\begin{array}{cc} 1.1a & b\\ 0.1a & 1.27a \end{array}\right)$	From FD transmiss.	3.51±0.61	**50.5**	**52.6**	$\left(\begin{array}{cc} 1.46 & 1.82\\ 0.13 & 1.69 \end{array}\right)$	3.5±2.0
10	$\left(\begin{array}{cc} a & a\\ 0 & a \end{array}\right)$	All equal, no m→f	3.07±0.19	**50.8**	**52.0**	$\left(\begin{array}{cc} 1.56 & 1.56\\ 0 & 1.56 \end{array}\right)$	1.7±0.5
5	$\left(\begin{array}{cc} a & 0\\ 0 & b \end{array}\right)$	No f↔m	4.58±1.61	**51.1**	**53.2**	$\left(\begin{array}{cc} 1.98 & 0\\ 0 & 2.33 \end{array}\right)$	5.1±1.2
7	$\left(\begin{array}{cc} a & a\\ b & b \end{array}\right)$	Equal reception	2.20±0.23	51.6	53.7	$\left(\begin{array}{cc} 1.47 & 1.47\\ 0.43 & 0.43 \end{array}\right)$	2.9±1.4
2	$\left(\begin{array}{cc} a & b\\ 0 & c \end{array}\right)$	no m→f	4.56±0.92	52.3	55.7	$\left(\begin{array}{cc} 1.02 & 3.61\\ 0 & 2.32 \end{array}\right)$	3.3±2.2
3	$\left(\begin{array}{cc} a & 0\\ b & c \end{array}\right)$	no f→m	3.20±0.38	53.0	56.3	$\left(\begin{array}{cc} 1.95 & 0\\ 0.15 & 1.63 \end{array}\right)$	4.1±1.5
4	$\left(\begin{array}{cc} a & b\\ b & c \end{array}\right)$	Equal f↔m	2.82±0.71	53.0	56.3	$\left(\begin{array}{cc} 1.87 & 0.20\\ 0.20 & 1.40 \end{array}\right)$	3.7±1.3
1	$\left(\begin{array}{cc} a & b\\ c & d \end{array}\right)$	None	4.00±9.11	54.3	59.2	$\left(\begin{array}{cc} 1.08 & 3.31\\ 0.09 & 1.95 \end{array}\right)$	3.5±9.1
9	$\left(\begin{array}{cc} a & a\\ a & a \end{array}\right)$	All equal	2.72±0.08	69.9	71.1	$\left(\begin{array}{cc} 0.94 & 0.94\\ 0.94 & 0.94 \end{array}\right)$	9.4±1.8
6	$\left(\begin{array}{cc} a & b\\ a & b \end{array}\right)$	Equal spread	3.56±1.17	71.2	73.3	$\left(\begin{array}{cc} 0.69 & 1.49\\ 0.69 & 1.49 \end{array}\right)$	9.6±2.0
11	$\left(\begin{array}{cc} a & 0\\ a & a \end{array}\right)$	All equal, no f→m	2.03±0.01	101	102	$\left(\begin{array}{cc} 1.03 & 0\\ 1.03 & 1.03 \end{array}\right)$	12±0.9

#### Empirical hypotheses about *F*

The values of *F* entries correspond to hypotheses about CWD transmission. These are formulated in [6]. Important components in CWD transmission may be sexual segregation (transmission within male and female social groups), between-group or within-mixed-group transmission, and transmission from females to males during mating. The four entries of *F* can be interpreted as characterizing the intensity of these mechanisms:

*F*_11_ (males to males)—transmission within male groups;
*F*_22_ (females to females)—transmission within female groups;
*F*_12_ (females to males)—combined transmission within mixed groups, between groups, and mating (rut) transmission from females to males;
*F*_21_ (males to females)—combined transmission within mixed groups and between groups;

We tested 10 types of matrix *F*, depending on 1, 2 and 3 parameters. The results are presented in Table 4 as models 2 to 11. We assume some of the entries are zero or equal. Column 2 shows the assumed type of the matrix *F* (template) with *a*,*b*,*c*,*d* being parameters to fit. Column 3 describes the corresponding hypothesis. Other columns show the details of the estimate. Formally, the best hypothesis is #8 with AIC = 49.4 and diagonal *F* with the same disease transmission both in males and females. However, five other cases cannot be totally rejected. Three models with the lowest AIC values have *R*_0_ equal to 3 or higher.

#### Deriving *F* from frequency-dependent disease transmission model

In [6] we have derived a number of expressions for force-of-infection terms corresponding to possible transmission paths for CWD that produce the observed 2:1 prevalence in males:females. The expressions for force of infection depend on population proportions, seasonality, transmission coefficients, and the disease prevalence. Assuming reasonable values for population parameters and linearizing the expressions with respect to prevalence, we obtain the entries of matrix *F* depending on two unknown transmission coefficients; see details in Appendix A3 in S1 File. It can be presented as a template with two parameters to fit, similar to empirical templates in lines 2 to 11 of Table 4. We tried six combinations of population parameters (see Appendix A3 in S1 File). Four of them gave close values of AIC, and one of the two models is shown in Table 4 in row 12. It appears second best in Table 4 with respect to AIC value.

#### Sensitivity of *R*_0_

For model 8, which has the lowest AIC and AIC_C_, the estimate of *R*_0_ is quite simple and allows us to see the sensitivity of the results to survival rates. Because the matrix *F* is diagonal, *R*_0_ = *F*_22_ *s*_*H*2_/(1 − *s*_*I*2_), provided the survival rates satisfy *s*_*H*2_/(1 − *s*_*I*2_) > *s*_*H*1_/(1 − *s*_*I*1_) (typically, survival rate for males is lower than for females [6]). Then, by differentiating with respect to *s*_*H*2_ and *s*_*I*2_, one obtains:

sensitivity: ∂*R*_0_ / ∂*s*_*H*2_ = *R*_0_ / *s*_*H*2_ ≈ 1.18*R*_0_, ∂*R*_0_ / ∂*s*_*I*2_ = *R*_0_/(1 − *s*_*I*2_) ≈ 2.78*R*_0_;
elasticity: ∂ln *R*_0_ / ∂ln *s*_*H*2_ = 1, ∂ln *R*_0_ / ∂ln *s*_*I*2_ = *s*_*I*2_ /(1 − *s*_*I*2_) ≈ 1.78.

Therefore *R*_0_ is sensitive to survival rates of both infected and uninfected females.

#### Which sex leaves more secondary infections?

**Fig 2:**
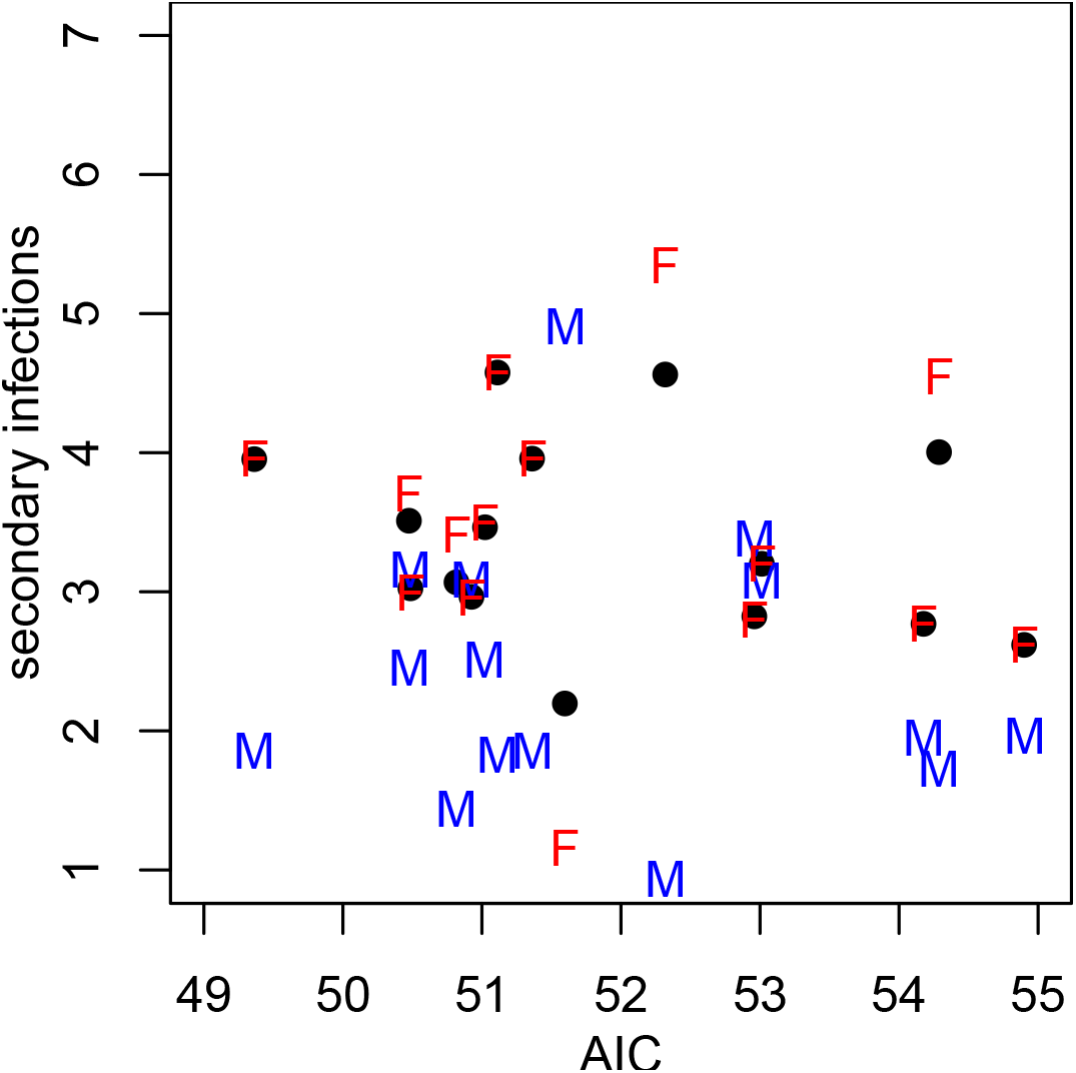
The number of secondary infections per one infected individual *R*_0_ (black circles), per one infected male *q*_*G*1_ (blue M) and per one infected female *q*_*G*2_ (red F) for buck:doe ratio 1:3. Most models predict that infected females create almost twice as many secondary infections than infected females. See Table 5 and Eq 10 for details.

The number of secondary infections per one male *q*_*G*1_ and per one female *q*_*G*2_ depend on population proportions, in our case on buck:doe ratio. Assuming buck:doe = 1:3 and 1:6, we calculated the values of *q*_*G*1_ and *q*_*G*2_ for all models in Table 4, and the results are presented in Table 5. In both cases, the four models with the lowest AIC have *q*_*G*2_ > *q*_*G*1_, which means that females create more secondary infections than males. For a buck:doe ratio of 1:3, *q*_*G*2_ is even greater than *R*_0_. Fig 2 shows the values of *R*_0_ plotted vs AIC for the best models with 49<AIC<55 (circles) with *q*_*G*1_ and *q*_*G*2_ for buck:doe = 1:3 denoted by letters M and F respectively.

**Table 5:** The number of new infections in the next generation (10) per one infected male *q*_*G*1_ and female *q*_*G*2_ for models in Table 4. Estimates are made for buck:doe ratio of 1:3 and 1:6. In the last column, there is management action giving the biggest reduction of secondary cases per one removed individual.

Model	AIC	*R*_0_	*q*_*G*1_, 3:1	*q*_*G*2_, 3:1	*q*_*G*1_, 6:1	*q*_*G*2_, 6:1	Primary removal
8	**49.4**	3.95	1.9	3.9	1.9	3.9	**Infected females**
12	**50.5**	3.51	1.6	2.4	1.9	4.1	**Infected females**
10	**50.8**	3.07	1.4	3.7	1.4	3.4	**Infected females**
5	**51.1**	4.58	1.8	4.6	1.8	4.6	**Infected females**
7	51.6	2.20	3.1	1.5	4.9	1.2	Infected males
2	52.3	4.56	0.94	6.13	0.94	5.34	Infected females
3	53.0	3.20	2.4	3.2	3	3.2	Infected females
4	53.0	2.82	2.6	2.8	3.4	2.8	~equal
1	54.3	4.00	1.4	5.3	1.7	4.5	Infected females
9	69.9	2.72	4.8	2.3	8.7	2.1	Infected males
6	71.2	3.56	3.5	3.6	6.4	3.2	~equal or males
11	101	2.03	5.2	2.0	9.5	2	Infected males

One explanation for this effect is higher survival rate for females: they live longer, have more time to transmit the disease to more individuals, and have more opportunity to infect more individuals through social and/or maternal behaviours. In addition, models 1, 2, 7, 10 and 12 in Table 4 support a higher transmission rate from females to males than vice versa. This may happen due to mating transmission and because of a higher proportion of females; see the derivation of the *F* template in Appendix A3 in S1 File.

This result means that removal of one infected female prevents more new infections than removal of one infected male. It may be one of the reasons why the lowest-AIC model in Table 3 accounts for the culling of females, but not males (*γ*_1_ = 0, *γ*_2_ > 0).

#### Conclusions

For all models in Table 4 with low AIC, we can see the following common features:

- there is strong disease transmission within each sex; therefore sexual segregation is important for disease transmission;
- there is no strong disease transmission from males to females;
- data cannot exclude strong disease transmission from females to males, because of the higher proportion of females and probably transmission during rut and doe interactions with male fawns and yearlings in the early social groups.

The estimates of *R*_0_ vary from 2.2 to 4.5 with most of the estimates exceeding 3.

## Discussion

In this paper, we develop a technique for estimating the basic reproduction number *R*_0_ from data on disease-prevalence dynamics in case of a structured population. *R*_0_ shows the mean number of secondary infections per one infected individuals. The disease spreads provided *R*_0_ > 1. On the other hand, if the disease-control measures (vaccination, removal of infected individuals etc.) can make *R*_0_ < 1, then the disease would not spread. For example, if *R*_0_ is obtained for the totally susceptible population, the disease would be stopped if proportion of immune individuals after vaccination exceeds 1 – *R*_0_^−1^.

There are several caveats to make against blind application of R0 analysis to disease outbreak [24]. For example, in the presence of a backward bifurcation it is possible that a disease will actually persist when *R*_0_ < 1, providing it is introduced at a sufficiently high level [3]. In this case, *R*_0_ > 1 is a sufficient condition for disease outbreak, but not a necessary condition. Furthermore, when there are multiple infection types, leading to cross infection dynamics, subtly different definitions for *R*_0_ (spectral radius of the next generation operator versus number of new infections arising from an initial infection of a particular type) can give different values for the quantity. Fortunately, both quantities cross the stability threshold *R*_0_ = 1 at the same parameter values. This leaves the utility of *R*_0_ as stability criterion unchanged, but brings into question how to interpret the concept of control when vaccination proportion exceeds 1 − *R*_0_^−1^. Here the key requirement is that the class of individuals vaccinated should then be correlated with the initial infection type.

The approach described in Section 2 is quite simple, and to the best of our knowledge has not been applied before to animal diseases with more than one infection compartment. However, as our results show, its implementation may encounter difficulties related to parameterizing the model because of a lack of data and necessity to estimate nuisance parameters like initial values of prevalence in Section 3. One more general problem is that growth of prevalence may be related mainly with the largest eigenvalue of *F*, and there may be many matrices with the same largest eigenvalue. This may create problems with estimability of all *F* entries and necessity for additional assumptions reducing the effective number of parameters. However, mechanistic models of disease transmission even with unknown parameters may still be helpful for interpreting the results and developing simplified templates for *F*.

When all types of individuals have close survival rates and disease transmission rates, then it may be simpler to use only one infected class and use classical methods for obtaining *R*_0_. However, for CWD in deer, this is not the case: males and females typically behave differently for a significant part of the year and have very different survival rates due to natural mortality and hunter preferences. The use of only one infected class may lead to an underestimation of the basic reproduction number.

In spite of some difficulties with obtaining error estimates for *R*_0_, our work provides new information about CWD spread among mule deer in Alberta. First, most *R*_0_ estimates are between 3 and 4, showing that CWD is quite contagious, so its control will be difficult without aggressive management. For example, if *R*_0_ = 3.5, then to stop the disease it is necessary to vaccinate more than 70% of the deer population, or the population should be harvested so intensely that the mean lifetime of deer decreases to less than one third.

Results of this paper agree with conclusions that were obtained in Potapov et al. [6] about the significant role of sexual segregation and transmission within deer bachelor and family groups. In spite of the observed higher prevalence in males compared to females, our analysis shows that infected females produce more secondary infections than males due to their higher survival rates and transmission to males during mating. This may mean that the major source of CWD spread may be female groups. If this disease pattern is close to reality, then male-only management harvest may reduce the number of infected individuals, but cannot stop the transmission of infection.

Estimates of model parameters related to the herd-reduction program in 2006–2008 show that removal of infected deer is efficient among females but not among males. The reason may be behavioural differences between males and females. The latter tend to stay within smaller areas, and the cull may cover a significant proportion of an infected female’s home range and stop the transmission among females. Indeed, Cullingham et al. [25] found that female deer harvested with 2 km were more genetically related than males, and CWD-positive deer were more likely to be related. In contrast, males move within larger areas than females [22,26], and if males contact individuals over a larger area they will increase the spatial spread of the disease.

On the other hand, the conclusions about the efficiency of culls in females (but not on the number of secondary infections left by females) are based mainly on the decline in estimated female prevalence between 2006 and 2007–2008. Therefore additional data are necessary to come to a more definite conclusion.

## Supporting Information

S1 File Appendix A1. Basic reproduction number for continuous-time model. Appendix A2. Likelihood for hunter-survey data. Appendix A3. Matrix *F* for a model of CWD transmission. (PDF)
