# Supplementary material for: Empirical Estimation of R 0 for Unknown Transmission Functions: The Case of Chronic Wasting Disease in Alberta
Source: PLoS One. 2015 Oct 9;10(10):e0140024. doi: 10.1371/journal.pone.0140024 (PMC4599850; doi:10.1371/journal.pone.0140024)
Supplement: S1 File — (PDF) [file pone.0140024.s001.pdf]

S1 File. Supporting Information

Appendix A1. Basic reproduction number for continuous-time model.

In the case of the continuous-time model, the equation governing the dynamics of $I_k(t)$ is

$$\frac{dI_k}{dt} = f(i_1,...,i_n)S_k - m_k I_k = S_{0k}\sum_{j=1}^{n} F_{kj} i_j - m_k I_k \ . \tag{A1}$$

Dividing it by S_{0k} we obtain the equation for the prevalence $i_k(t)$,

$$\frac{di_k}{dt} = \sum_{j=1}^{n} F_{kj} i_j - m_k i_k \ . \tag{A2}$$

Like in case of discrete-time model, this equation allows us to fit F from the data on change in the observed disease prevalence. Again, to estimate the basic reproduction number, we need an equation in terms of individuals rather than prevalence. To connect these two quantities we introduce the diagonal matrices of both numbers of susceptible individuals and mortality rates

$$D = \mathrm{diag}(S_{01},...,S_{0n}), \qquad V = \mathrm{diag}(m_1,...,m_n) \tag{A3}$$

and use the same relations as in discrete-time case, $I = Di$ or $i = D^{-1}I$. Then i can be excluded from Eq. (2), which in matrix form becomes

$$dI/dt = DFD^{-1}I - VI \ . \tag{A4}$$

The basic reproduction number R_0 is the spectral radius $\rho(\cdot)$ or the maximum eigenvalue of the next-generation matrix G,

$$R_0 = \rho(G), \qquad G = DFD^{-1}V^{-1}; \tag{A5}$$

see [22]. However, it is simple to show that eigenvalues of the matrices $M = FV^{-1}$ and G coincide, and hence

$$R_0 = \rho(M), \qquad M = FV^{-1}. \tag{A6}$$

The matrix of marginal force of infection F can be estimated from data by fitting the model Eq. (3), while mortality rates are to be obtained elsewhere.

Equivalence of continuous- and discrete-time approaches. For small time step τ, it is possible to use the relation between mortality rate and per time step survival as $s_k = \exp(-\tau m_k)$, then approximately $1 - s_{Ik} \approx \tau m_k$, so $\tau(1 - S_I)^{-1} \approx V^{-1}$, $s_{Hk} = \exp(-\tau m_{Hk}) \approx 1$, after which (7) and (9) turn into (A5) and (A6). Therefore, both continuous- and discrete-time approaches are equivalent, and the choice of discrete- or continuous-time model is a matter only of convenience.

Appendix A2. Likelihood for hunter-survey data.

Here is the derivation of the likelihood expressions. We assume that there is a population of *N* deer, *n* deer are selected (hunted) and *k* of them are CWD positive. If true prevalence is *i*, then the mean number of positive is *in*, and, assuming a Poisson distribution for *k*, we have the probability to have exactly *k* animals:

$$P_k = \frac{(in)^k}{k!}\exp(-in).$$

If we have a sequence of data in the form $n(t), k(t)$, as in Table 1, and a model for $i(t)$, then the log-likelihood of these data is

$$L = \sum_t \left(k(t)\log(i(t)n(t)) - i(t)n(t) - \log(k(t)!)\right).$$

Appendix A3. Matrix F for a model of CWD transmission

In Potapov et al. [6], a model for CWD transmission has been developed with multiple transmission mechanisms. One of the combinations considered as plausible included transmission within social groups and female-to-male transmission during rut due to some features of male mating behaviour. The model includes also juveniles, but we assume here that juveniles are always healthy and get the disease only becoming adults. The corresponding expressions for the force of infection are adapted to notation of this paper. Again, index 1 corresponds to males, 2 to females:

$$f_1 = \beta\left(w_S \frac{I_1}{N_1} + w_M \frac{I}{N} \right) + \beta_R \frac{I_2}{N_1},$$

$$f_2 = \beta\left(w_S \frac{I_2}{N - N_1} + w_M \frac{I}{N} \right).$$

The first term in brackets corresponds to sexually segregated groups (males and females with juveniles were considered as two separate groups), the second one corresponds to winter mixed groups; w_S, w_M are the weights showing contribution of each group types into overall disease transmission, β, β_R are transmission coefficients, N_1, N_2, N are population size or density of adults of the corresponding sex and the total population. Substituting $I_1 = N_1 i_1, I_2 = N_2 i_2$, $I = I_1 + I_2$ and denoting proportion of adult males $\pi_1 = N_1 / N$, females $\pi_2 = N_2 / N$, and the ratio of the weights $g = w_M / w_S$, we obtain

$$f_1 = \beta w_S \left(i_1 + g\left(\pi_1 i_1 + \pi_2 i_2 \right) \right) + \beta_R \frac{\pi_2}{\pi_1} i_2,$$

$$f_2 = \beta w_S \left(\frac{\pi_2}{1 - \pi_1} i_2 + g\left(\pi_1 i_1 + \pi_2 i_2 \right) \right).$$

Therefore

$$F_{11} = \beta w_S \left(1 + \pi_1 g \right),$$

$$F_{12} = \beta w_S \pi_2 g + \beta_R \left(\pi_2 / \pi_1 \right),$$

$$F_{21} = \beta w_S \pi_1 g,$$

$$F_{22} = \beta w_S \left(\pi_2 \left(1 - \pi_1 \right)^{-1} + \pi_2 g \right).$$

At present, there are no data about the values of transmission coefficients. For the ratio of the seasonal weights, according to [6], it is likely that $g \le 1$, so we tried values $g = 1$, 0.5, and 0. Population proportions may strongly vary in space and time, but reasonable values to test may be 20% of adult males and 50% of adult females (many males) and 10% of adult males and 60% of adult females (few males); that is, $\pi_1 = 0.2, \pi_2 = 0.5$ and $\pi_1 = 0.1, \pi_2 = 0.6$. For fitted values, it is convenient to take $a = \beta w_S$ and $b = F_{12}$, which gives the following template for *F*:

$$F = \begin{pmatrix} (1+\pi_1 g)a & b \\ \pi_1 g a & \left(\frac{\pi_2}{1-\pi_1} + \pi_2 g\right)a \end{pmatrix}.$$

The results for six combinations of π_1, π_2, g are shown in Table A1. The model F4 for $\pi_1 = 0.1, \pi_2 = 0.6, g = 1$ is model 12 in Table 4. The basic reproduction numbers for all six models are shown in Fig. 2.

Table A1. Matrix *F* derived from the model of frequency-dependent CWD transmission..

#	*F* type	Hypothesis on transmission	R_0 ±se	$-\log L$	AIC	*F*	$\gamma_2 \times 10^{-4}$
F4	$\begin{pmatrix} 1.1a & b \\ 0.1a & 1.27a \end{pmatrix}$	$g=1, \pi_1 = 0.1,$ $\pi_2 = 0.6$	3.51±0.61	21.24	**50.5**	$\begin{pmatrix} 1.46 & 1.82 \\ 0.13 & 1.69 \end{pmatrix}$	3.5±2.0
F3	$\begin{pmatrix} 1.2a & b \\ 0.2a & 1.12a \end{pmatrix}$	$g=1, \pi_1 = 0.2,$ $\pi_2 = 0.5$	3.03±0.41	21.24	**50.5**	$\begin{pmatrix} 1.37 & 2.15 \\ 0.23 & 1.28 \end{pmatrix}$	3.5±1.7
F5	$\begin{pmatrix} 1.1a & b \\ 0.1a & 0.88a \end{pmatrix}$	$g=0.5, \pi_1 = 0.2,$ $\pi_2 = 0.5$	2.97±0.37	21.46	50.9	$\begin{pmatrix} 1.84 & 0.37 \\ 0.17 & 1.47 \end{pmatrix}$	3.4±2.1
F6	$\begin{pmatrix} 1.05a & b \\ 0.05a & 0.97a \end{pmatrix}$	$g=0.5, \pi_1 = 0.1,$ $\pi_2 = 0.6$	3.47±0.59	21.51	51.0	$\begin{pmatrix} 1.90 & 0.24 \\ 0.09 & 1.75 \end{pmatrix}$	3.7±2.4
F2	$\begin{pmatrix} a & b \\ 0 & 0.67a \end{pmatrix}$	$g=0, \pi_1 = 0.1,$ $\pi_2 = 0.6$ (no mixed groups)	2.77	23.09	54.2	$\begin{pmatrix} 2.12 & 0.00 \\ 0 & 1.41 \end{pmatrix}$	1.9±0.4
F1	$\begin{pmatrix} a & b \\ 0 & 0.62a \end{pmatrix}$	$g=0, \pi_1 = 0.2,$ $\pi_2 = 0.5$ (no mixed groups)	2.62	23.45	54.9	$\begin{pmatrix} 2.13 & 0.00 \\ 0 & 1.33 \end{pmatrix}$	1.6±0.4

For the model F3, $g = 1, w_S = w_M = 1$, $a = \beta = 1.14 \text{year}^{-1}$, $b = 2.15 \text{year}^{-1}$, $\beta_R = (b - a\pi_2 g)\pi_1 / \pi_2 = 0.27 \text{year}^{-1}$. For the model F4, $g = 1, w_S = w_M = 1$,

$\beta = 1.33 \text{year}^{-1}$, $b = 1.82 \text{ year}^{-1}$, $\beta_R = 0.1 \text{year}^{-1}$. For models F1 and F2, estimate of standard error failed due to the degeneracy of the Hessian.
